# Disorder Tolerance in LED Phosphor Host CaAlSiN_3_: Correlated Cation Disorder with Minimal Electronic Penalty

**DOI:** 10.1021/acs.chemmater.6c00669

**Published:** 2026-07-07

**Authors:** Maryia Shymanovich, Alexander G. Squires, Jakoah Brgoch, David O. Scanlon

**Affiliations:** † School of Chemistry, 1724 University of Birmingham, Birmingham B15 2TT, U.K.; ‡ Department of Chemistry, 14743 University of Houston, Houston, Texas 77204, United States; § Texas Center for Superconductivity, 14743 University of Houston, Houston, Texas 77204, United States

## Abstract

In functional multinary nitrides, intrinsic cation disorder typically has severe electronic consequences, introducing gap states and driving band gap reduction through local bonding irregularities. CaAlSiN_3_ (CASN), a benchmark nitride phosphor host for phosphor-converted light-emitting diodes, therefore presents a paradox: despite substantial Al/Si disorder generated during high-temperature synthesis, it maintains its exceptional optical performance. Understanding the fundamental effects of intrinsic disorder in this system could therefore provide broader insight into both its performance as a phosphor and the electronic structure of disorder-tolerant nitrides. Experiments typically treat the Al/Si disorder in CASN as random, whereas prior theoretical studies have been limited to a narrow set of ordered configurations, leaving its thermodynamic origin and electronic consequences unresolved. Here, we combine cluster-expansion Monte Carlo simulations with hybrid density functional theory to characterize the Al/Si disorder in CASN across a range of temperatures. Despite the emergence of disorder, the electronic structure remains resilient, with a maximum band gap reduction of only 3.8% and minimal carrier localization. This contrasts with related ternary nitrides, such as ZnGeN_2_ and ZnSnN_2_, in which less correlated cation disorder directly perturbs both band edges, driving pronounced band gap collapse and carrier localization. We attribute this tolerance to two features of CASN’s crystal chemistry: a Ca d-dominated conduction-band minimum decoupled from the disordered sublattice and correlated disorder that suppresses the most electronically damaging local environments. These results establish CASN as a model system for disorder tolerance in multinary nitrides and suggest broader design principles for electronically robust phosphor hosts.

## Graphical abstract

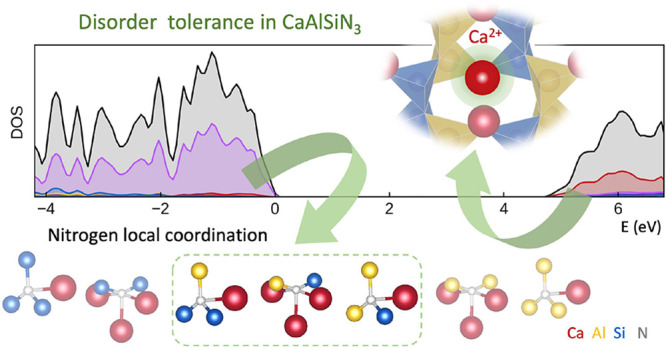

## Introduction

The widespread adoption of light-emitting diode (LED)-based solid-state lighting offers a significant opportunity to reduce global electricity consumption and associated carbon emissions.^1,2^ Among the most successful device architectures for general white lighting are phosphor-converted LEDs (pc-LEDs), in which blue or near-ultraviolet emission from an LED chip is partially converted to longer wavelengths by an inorganic phosphor, producing broadband white light.^3,4^ Within this landscape, CaAlSiN_3_ (CASN) has emerged as a benchmark nitride phosphor host: when doped with Eu^2+^ or Ce^3+^, it exhibits high efficiency, thermally robust long-wavelength emission under blue excitation, and has been widely implemented in high color-rendering warm-white pc-LEDs.^5–7^

Yet CASN harbors a poorly understood structural feature: Al and Si share the same tetrahedral sites within its orthorhombic framework of corner-sharing [AlN_4_] and [SiN_4_] tetrahedra, inherently enabling extensive intrinsic cation disorder within the host sublattice (Figure 1). Experimental studies have consistently reported substantial Al/Si disorder, most commonly described as “random” based on X-ray diffraction measurements.^8,9^ Though the similar X-ray scattering factors of Al and Si render conventional diffraction largely insensitive to their relative distributions, making it difficult to distinguish true random disorder from short-range correlated order.^10^ In other multinary nitrides, intrinsic cation disorder of this kind is electronically devastating: in ZnSnN_2_ and ZnGeN_2_, for instance, it drives severe band gap reduction, carrier localization, caused by electrostatic potential fluctuations.^11–15^ That CASN performs so well despite supposedly random cation disorder is far from obvious.

**Figure 1:**
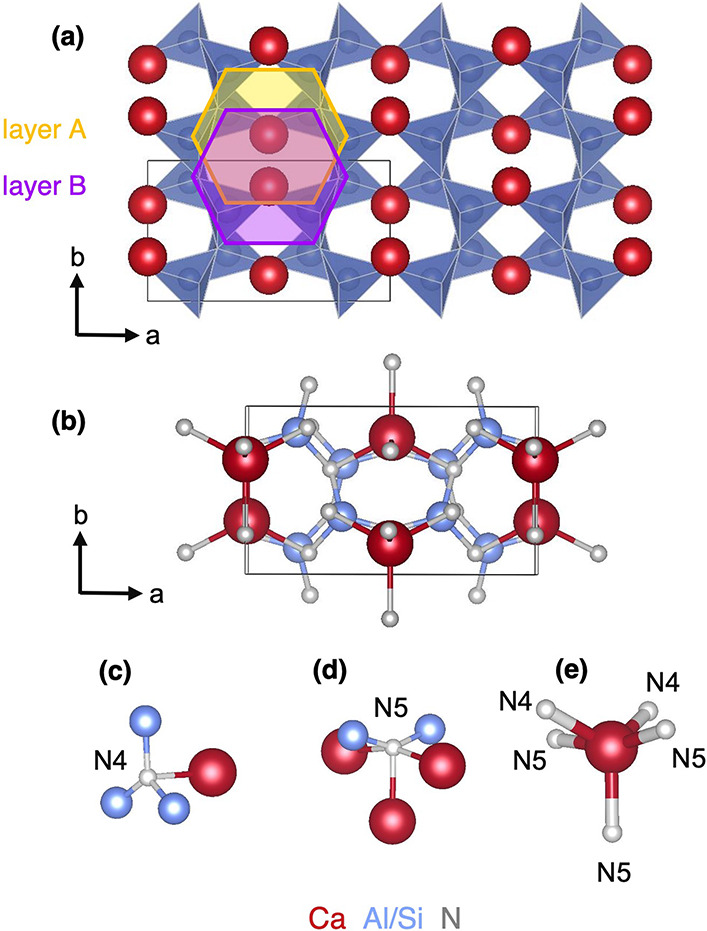
Crystal structure of CASN in a–b plane. Al and Si atoms occupy the same tetrahedral sites. (a) Layers A and B formed by six-membered rings. Ca atoms displayed here are in the centers of rings of a layer B. (b) 24-atom unit cell of CASN containing 8 Al/Si sites. Panels (c–e) show coordination environments of N4, N5, and Ca atoms, respectively. Ca, red; Al/Si, light-blue; and N, gray.

The effects of disorder on phosphor performance have been studied extensively, but almost exclusively from the perspective of the activator site: perturbation of the local crystal-field environment around Eu^2+^ or Ce^3+^ by the surrounding host is well-established as a route to nonradiative losses and thermal quenching.^16,17^ Intrinsic disorder within the host sublattice itself—remote from the activator—has received far less attention, and its consequences for electronic structure and optical performance remain poorly understood. CASN is therefore a uniquely interesting case: anomalous among nitrides for its apparent tolerance to intrinsic cation disorder, and representative of a broader blind spot in phosphor physics.

Several first-principles studies have sought to address cation ordering in CASN, but have been limited to a small number of idealized ordered configurations.^18–20^ These cannot capture the thermodynamic ensemble of configurations accessible at synthesis temperatures (1700–2100 K), nor assess how correlated disorder influences the electronic structure. The microscopic nature of Al/Si disorder in CASN—whether it is truly random, correlated, or locally ordered—and its implications for electronic performance therefore remain unresolved.

Here, we address this by combining cluster-expansion Monte Carlo simulations—commonly used to resolve correlated disorder in functional materials^21–25^ —with hybrid density functional theory to investigate Al/Si disorder in CASN as a function of temperature. We identify an order–disorder transition near 850 K, above which CASN adopts a strongly correlated disordered phase consistent with synthesis conditions. Despite pronounced Al- and Si-rich local coordination motifs, the electronic structure remains resilient: the band gap narrows by at most 3.8% and carrier localization is minimal—in stark contrast to ZnSnN_2_ and ZnGeN_2_, where less correlated disorder directly perturbs both band edges. We attribute this tolerance to two features of the crystal chemistry of CASN acting in concert: a conduction-band minimum dominated by Ca *d* states that is decoupled from the disordered sublattice, and a strongly correlated disorder regime that suppresses the most electronically damaging local environments. Together, these results establish CASN as a model system for correlated disorder in multinary nitrides and identify design principles for electronically robust phosphor hosts.

## Computational Methods

### DFT Calculations

DFT^26,27^ calculations were performed using Vienna ab initio simulation package (VASP).^28–31^ To construct the cluster-expansion model, the energies of different Al/Si orderings were obtained from geometry optimization using Perdew–Burke–Ernzerhof (PBE) generalized gradient approximation (GGA) functional revised for solids (PBEsol).^32^ The initial geometries for optimization are based on experimental results obtained by Uheda et al.^8,33^ Selected low-energy orderings from the cluster-expansion training set were further relaxed and characterized using the Heyd–Scuseria–Ernzerhof (HSE06) screened hybrid-DFT functional.^34,35^ The energetic ordering of the structures was found to be insensitive to the choice of functional (Figure S5). The HSE06 functional was used to perform further electronic-structure calculations for the two lowest-energy orderings and the disordered supercells. In all calculations, a plane-wave cutoff energy of 700 eV and a Γ-centered Monkhorst–Pack^36^ k-mesh with 0.32 Å^–1^ k point-spacing were used. For the geometry relaxations, the cutoff energy was increased by 30*%* (910 eV) to avoid Pulay stress effects. Both the energy cutoff and k-spacing stated above were obtained from total-energy convergence tests performed for 24-atom unit cell structure of CASN with a randomly chosen cation ordering (space group *Pmc2*_*1*_ (26)). Following band-structure results, denser *k*-meshes (0.23 Å^–1^) were used for some structures in density-of-states (DOS) calculations to obtain accurate band edges, as DOS data were required for subsequent inverse participation ratio analysis. Tolerances of 10^–6^ eV and 10^–2^ eV/Å were applied to electronic and ionic convergence, respectively, for all calculations. All DOS and electronic band structures were plotted with *sumo*.^37^ Gaussian smearing of 0.05 eV was applied in-post to DOS plots. The values of band gap and band edges were obtained from *sumo*. Band edges were aligned using average values of Ca 1*s* core state eigenvalues as inert reference states. All structures were visualized using *VESTA*.^38^ The results from VASP were analyzed using *Python Materials Genomics (pymatgen)* package.^39,40^ Madelung site potential energy was computed using Ewald technique^41^ for periodic systems as implemented in *pymatgen*.

### Cluster-Expansion Fitting

The cluster-expansion (CE) model was trained using DFT-calculated energies for 133 CASN structures containing 24, 36, 48, and 96 atoms with different Al/Si orderings in a 1:1 ratio. The initial structures were generated by enumeration using *Integrated Cluster Expansion Toolkit (ICET)* ^42–45^ from the 12-atom primitive cell. The energy CE model was fit using *trainstation* ^46,47^ with a final cross validation (CV) error of 5.5 meV/atom. Among the fitting methods tested before CE construction, the automatic relevance detection regression (ARDR) method achieved the best performance. The final CE model includes pair clusters only with pair cutoff radius of 6.0 Å. Further details of the CE model’s construction and validation are provided in the Supporting Information.

### Monte Carlo simulations

Monte Carlo (MC) simulations were carried out using the *mchammer* module of *ICET* under the canonical ensemble for a 1536-atom supercell over the temperature range of 100–2200 K with 50 K step. A supercell of *Cc* ground-state structure was used to initialize the simulation at 100 K. For subsequent temperatures, the final equilibrated configuration obtained at temperature *T*K was used as the starting structure for the simulation at *T* + 50 K. Simulations were run for 120,000 cycles, with 1 cycle comprising 512 steps (the number of steps required to swap each cation), including 8000 equilibration cycles. Representative structures matching the average cluster vectors obtained from MC simulations at selected temperatures were generated.

## Results

### Order–Disorder Transition and Short-Range Order

The finite-temperature evolution of Al/Si ordering in CaAlSiN_3_ was investigated using Monte Carlo simulations based on the fitted cluster-expansion model. The average energy and heat capacity between 100 and 2200 K are shown in Figure 2. The heat capacity displays a sharp peak at 850 K, identifying a first-order order–disorder phase transition. Below this transition, the lowest-energy configuration has space group *Cc* (no. 9), in which [AlN_4_] and [SiN_4_] tetrahedra form “double layers” along the *c* direction, perpendicular to planes of hexagonal rings (Figure 3a,b). The second-lowest-energy configuration, with space group *P2*_*1*_ (No. 4), lies within 1 meV/atom of the ground state and adopts a characteristic zigzag arrangement along *c* (Figure 3c,d). In both polymorphs, the hexagonal rings are composed of alternating Al- and Si-centered tetrahedra. Lattice parameters and relative energies are summarized in Table 1.

**Figure 2:**
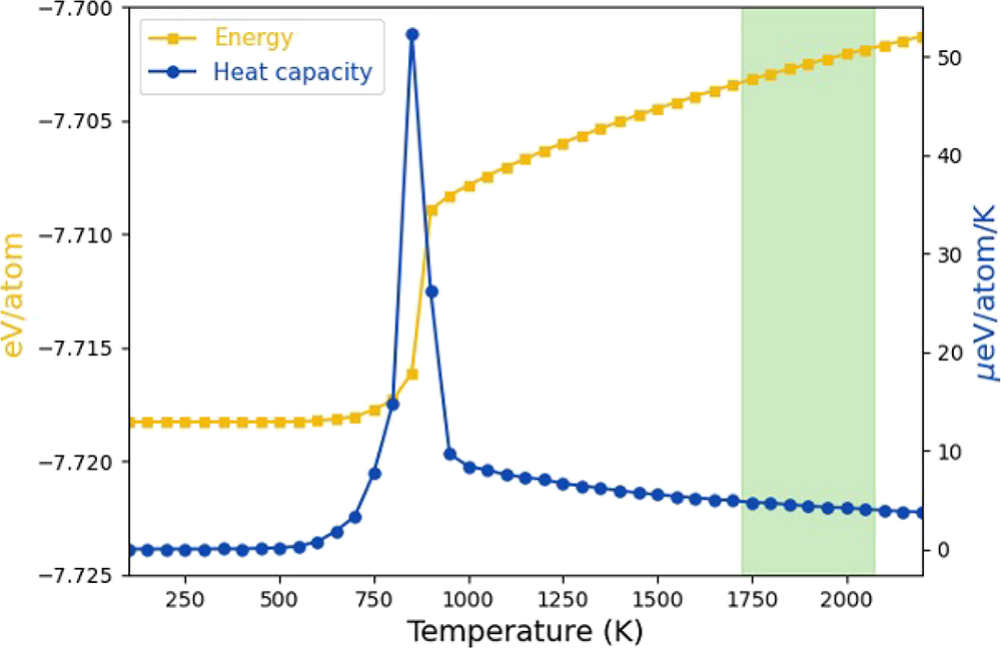
Change in average energy and heat capacity from Monte Carlo simulations with respect to temperature. The green region shows the range of synthesis temperatures (high-T synthesis methods only).

**Figure 3:**
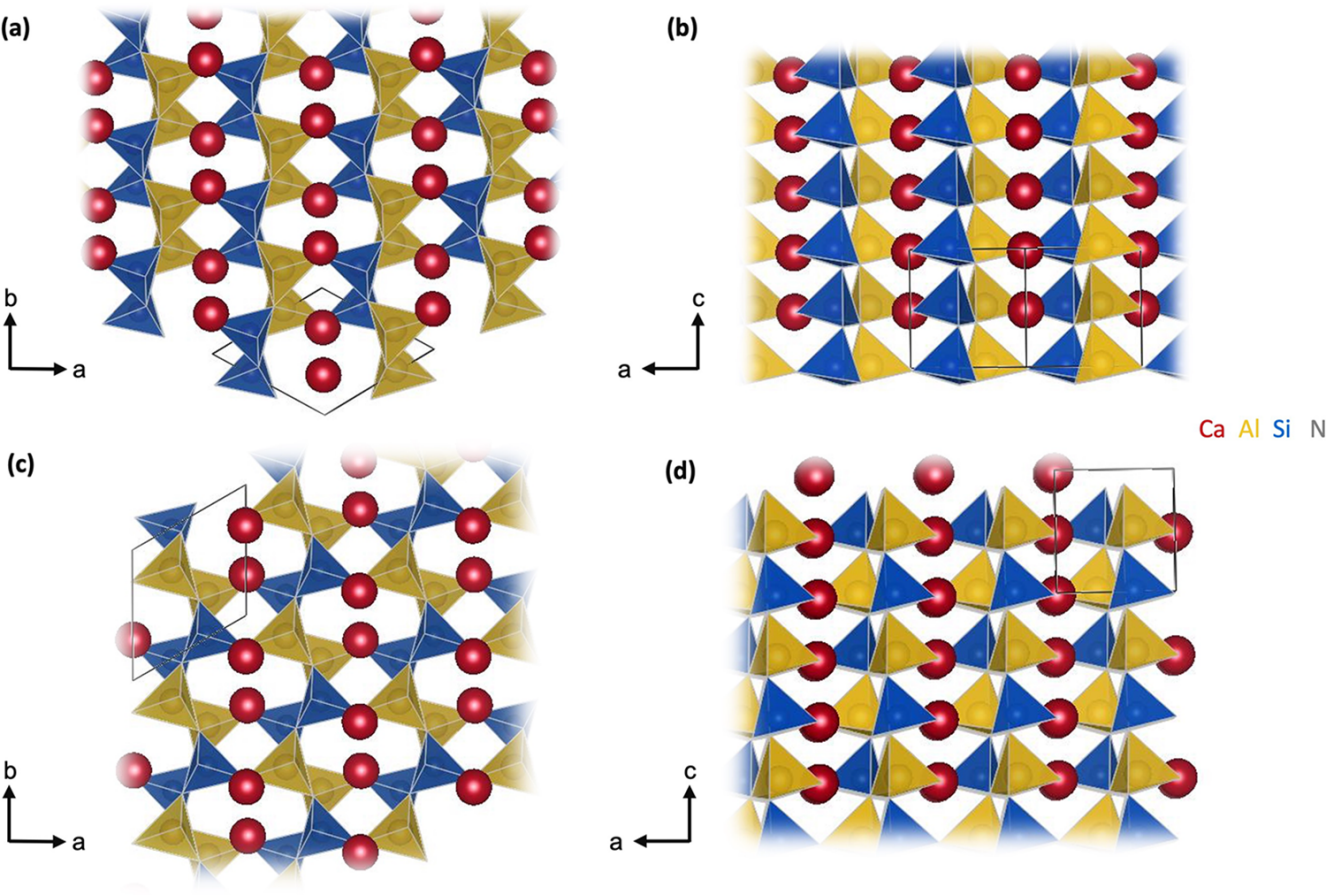
(a,b) and (c,d) show cation (tetrahedra) arrangements in *Cc* (9) and *P2*_*1*_ (4), respectively, in a-b and a-c planes. Ca, red; Al, yellow; Si, blue; and N, gray. Black frames show the 12-atom primitive cells.

**Table 1:** Relative Energies and Lattice Parameters of *Cc* (9) and *P2*_*1*_ (4), Obtained with PBEsol and HSE06 Functionals and Compared with Previous Theoretical and Experimental Data

	Rel. energy (meV/atom)		PBEsol lattice parameters (Å)			HSE06 lattice parameters rel. to PBEsol(Å)			Mikami et al.^18^ DFT-PBE rel. to PBEsol (Å)		
symmetry/space group	PBEsol	HSE06	*a*	*b*	*c*	*a*	*b*	*c*	*a*	*b*	*c*
*Cc* (9)	0	0	9.8692	5.6539	5.0372	–0.0205	–0.0063	–0.0014	+0.042	+0.0348	+0.0329
*P2*_*1*_ (4)	+0.16	+0.22	9.8646	5.6537	5.0385	–0.0207	–0.0056	–0.0012	+0.0468	+0.0302	+0.0341
			Exp. data for Cm2_1_ (36)								
Uheda et al.^8,33^			9.8020	5.6506	5.0633						
Piao et al.^9^			9.7311	5.6408	5.0417						

Short-range order (SRO) parameters were calculated following the Warren–Cowley formula,^48,49^

$$\alpha_{ij}^{\left(r\right)}=1-\frac{P_{ij}^{\left(r\right)}}{c_{i}c_{j}}$$ 1

where *P*_*ij*_^(*r*)^ is the probability of finding atoms *i* and *j* at distance *r*, and *c*_*i*_, *c*_*j*_ are their concentrations. α = 0 corresponds to a random distribution, α > 0 to clustering of like species, and α < 0 to preferential unlike-pair ordering.

Figure 4 shows the SRO parameters for the first seven pair clusters. Below 600 K, the parameters are consistent with the *Cc* ground state. At 850 K they drop abruptly, confirming the phase transition. Above the transition, short-range correlations persist strongly across all synthesis-relevant temperatures: the parameter for cluster 4 remains as negative as −0.4 at 2000K, indicating a robust preference for unlike Al–Si pairs at that distance, while the three shortest-range parameters stabilize near −0.2. Only the longest-range parameters approach zero. The disordered phase above 850 K is therefore not random but retains pronounced short-range correlations.

**Figure 4:**
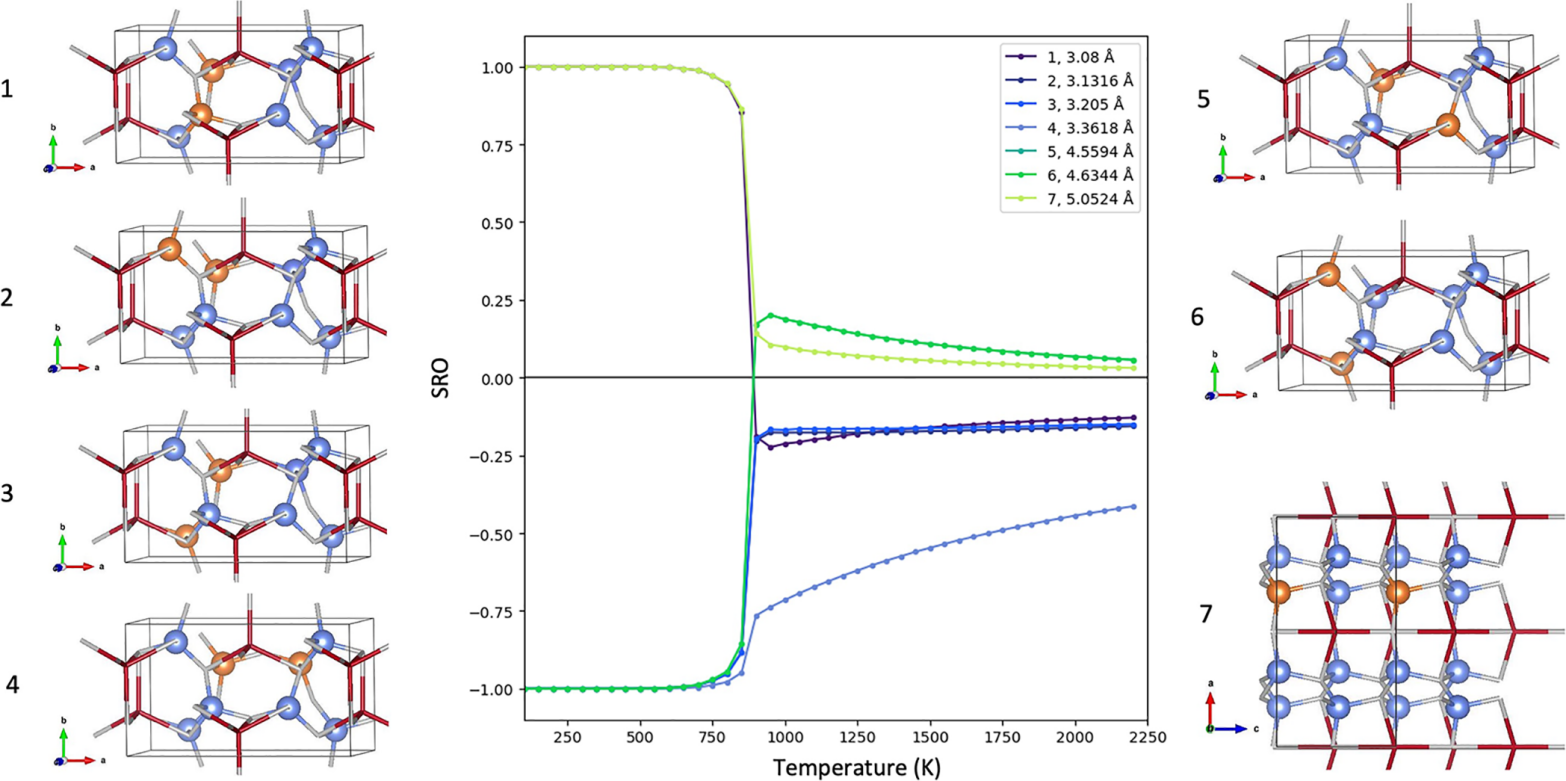
Variation of short-range order parameter for the first seven pair clusters (indexed 1–7 on Figure S5) with respect to Monte Carlo temperature. Ca and N atoms are not shown. Cation sites corresponding to each cluster pair are shown in orange. Cation–cation distances are given in the box.

Since N atoms are directly bonded to Al and Si, their local coordination provides a complementary short-range order descriptor, as used in related nitride studies.^11,12,50^ The two symmetry-inequivalent N sites—N4 (coordinated by 1 Ca and 3 Al/Si) and N5 (coordinated by 3 Ca and 2 Al/Si)—give rise to seven possible local environments, shown in Figure 5 along with their Pauling bond strength sums calculated using Pauling’s electrostatic valence rule.^51^ The ground-state motifs (N4–21, N4–12, N5–11) have bond strength sums close to 3+, while the Pauling-rule-violating motifs (N4–30, N4–03, N5–20, N5–02) deviate by up to ± 0.4.

**Figure 5:**
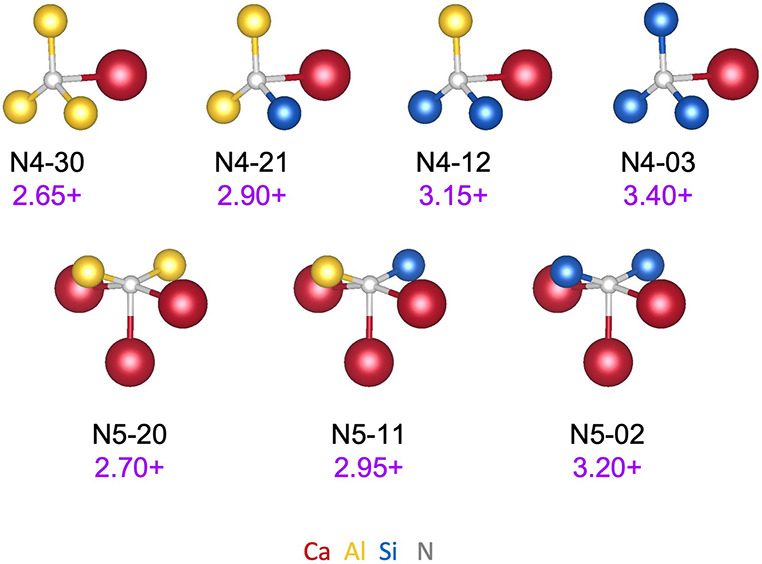
Possible nitrogen local environments (“motifs”) in CaAlSiN_3_. Bond strength sum at the N anion from adjacent cations, calculated using Pauling’s valence bond strength rule, is shown in purple. Ca, red; Al, yellow; Si, blue; and N, gray.

Motif fractions extracted from Monte Carlo simulations are shown in Figure 6. Below 650 K, all N atoms occupy ground-state environments exclusively. The phase transition at 850 K is accompanied by an abrupt increase in the Pauling-rule-violating motifs, but their populations remain limited even at synthesis temperatures: at 2000 K, over 80% of N4 atoms and 70% of N5 atoms retain their ground-state environments. This confirms that the high-temperature disordered phase of CASN is correlated rather than random, with the most electrostatically unfavorable motifs suppressed by the underlying short-range ordering preference.

**Figure 6:**
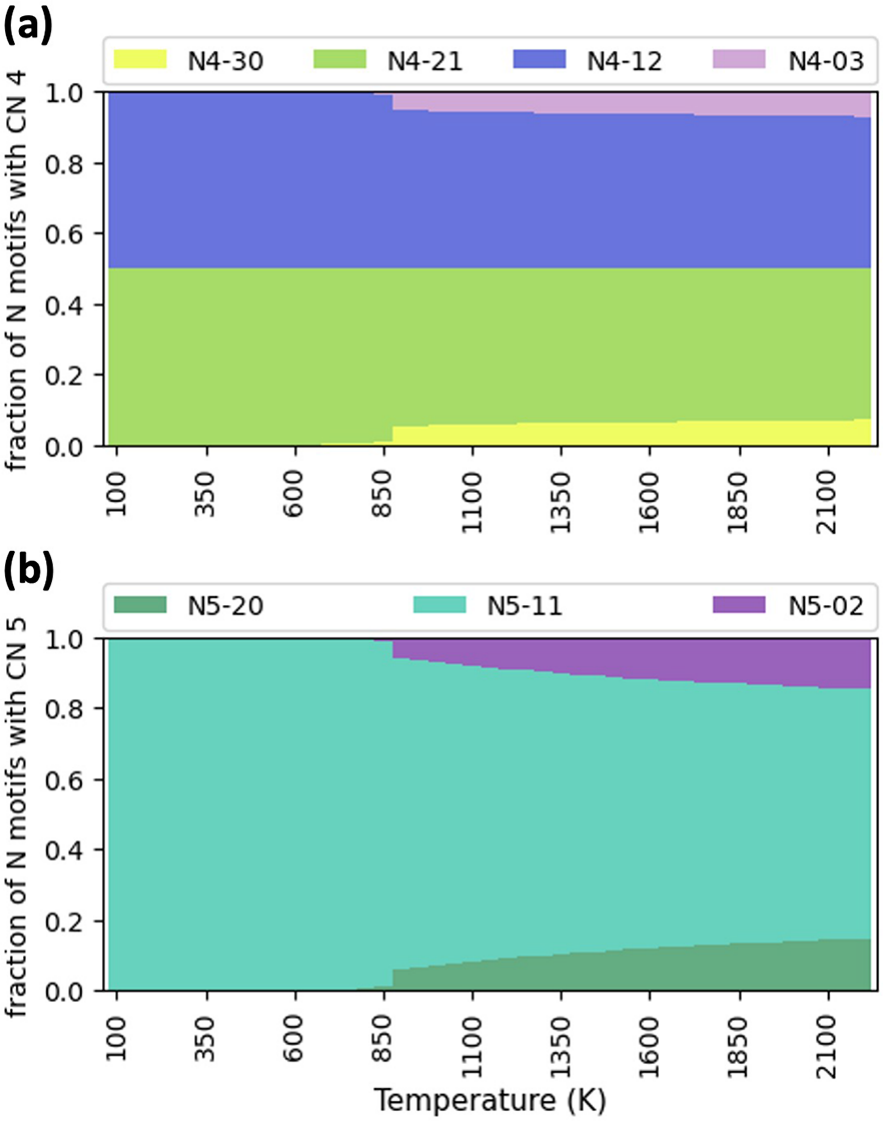
Average fractions of N motifs as a function of temperature from Monte Carlo simulations, obtained from 1000 randomly chosen supercells for (a) N4 and (b) N5 atoms.

### Electronic Structure

The electronic structures of the *Cc* and *P2*_*1*_ ordered polymorphs, examined using HSE06, serve as the reference point for assessing the impact of disorder. Both exhibit an indirect band gap of 4.73 eV (Figures 7 and S9), with the CBM at Γ and the VBM along the Γ–*A* and Γ–*Z* paths, respectively. The calculated fundamental gaps are slightly smaller than experimentally reported values of 5.0–5.2 eV,^8–10,33,52–55^ though optical gaps extracted from absorption spectra (Figures S7 and S8) exceed 5.0 eV, consistent with the expected difference between fundamental and optical gaps. Prior GGA calculations gave significantly smaller gaps (≈3.4 eV),^18–20^ reflecting the well-known underestimation by semilocal functionals.^56^ Projected densities of states show that the VBM is dominated by N *p* states and the CBM by Ca *d* states—a distinction that proves central to understanding the electronic response to disorder. Both ordered polymorphs share identical N coordination environments (N4–21, N4–12, and N5–11 only), consistent with their identical band gaps and confirming that long-range cation arrangement has minimal electronic consequence when preferred short-range order is preserved.

**Figure 7:**
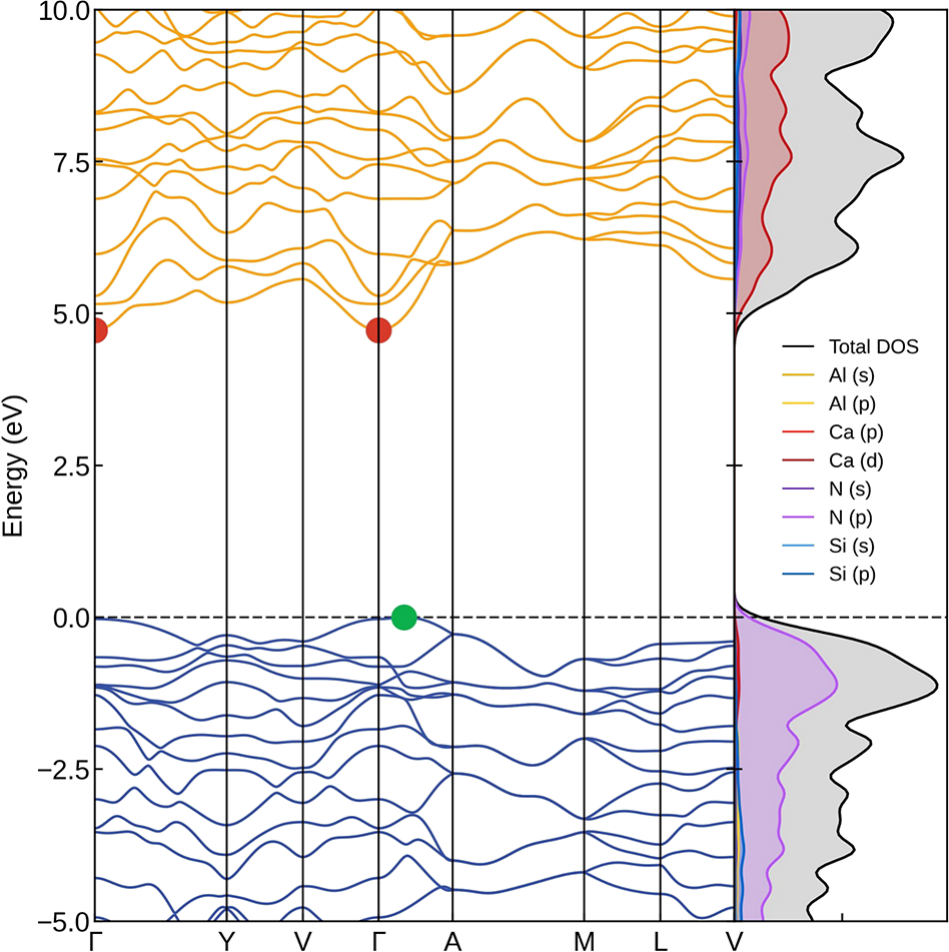
Band structure and density-of-states of *Cc* (9) ordered structure. VBM is set as zero.

To assess the effect of disorder on electronic properties, screened hybrid calculations were performed on a series of 144-atom representative supercells (Table 2). The first two, D1 and D2, were obtained by swapping one Al–Si pair within an ordered supercell, representing the onset of disorder over the 600–800 K range. Three further supercells were generated by matching average cluster vectors obtained from Monte Carlo simulations at 900 K (just above the phase transition), 1700, and 2000 K, the latter two lying within the synthesis temperature range. Table S3 provides a comparison of average cluster vectors obtained from MC simulations and cluster vectors of representative structures.

**Table 2:** Relative Energies and N Motif Concentrations for 144-Atom Supercells

		fraction (number) of N4 motifs				fraction (number) of N5 motifs		
structure	DFT-HSE06 Rel. energy (meV/atom)	N4–30	N4–21	N4–12	N4–03	N5–20	N5–11	N5–02
*Cc* (9)	0		0.500 (24)	0.500 (24)			1.000 (24)	
D1	+3.02	0.021 (1)	0.479 (23)	0.479 (23)	0.021 (1)		1.000 (24)	
D2	+4.06	0.021 (1)	0.458 (22)	0.521 (25)		0.042 (1)	0.917 (22)	0.042 (1)
900 K	+17.08	0.063 (3)	0.438 (21)	0.438 (21)	0.063 (3)	0.125 (3)	0.750 (18)	0.125 (3)
1700 K	+20.29	0.083 (4)	0.396 (19)	0.458 (22)	0.063 (3)	0.125 (3)	0.750 (18)	0.125 (3)
2000 K	+21.41	0.104 (5)	0.354 (17)	0.479 (23)	0.063 (3)	0.125 (3)	0.750 (18)	0.125 (3)

The electronic band gaps and band edges are shown in Figure 8. Single antisite pairs (D1, D2) have negligible electronic effect, with the band gap of D2 decreasing by only 0.02 eV. The transition to the disordered state produces a small but progressive reduction: from 4.73 eV in the ground state to 4.63 eV at 900 K, 4.60 eV at 1700 K, and 4.55 eV at 2000 K. The reduction arises almost entirely from an upward shift of the VBM; the CBM position is unchanged across all supercells, shifting by at most 0.01 eV.

**Figure 8:**
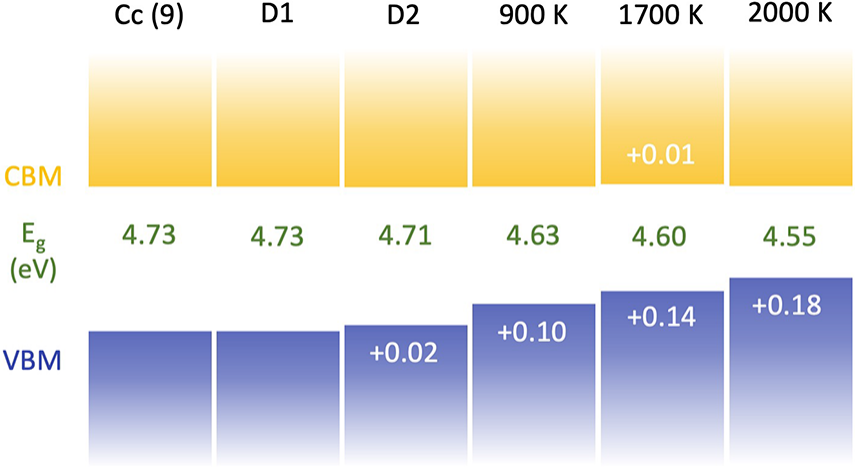
Schematic representation of relative band-edge positions and band gap sizes for the representative supercells. Changes (eV) in VBM and CBM with respect to the *Cc* ordering are shown in white. Band gap values are given in green. Band edges are aligned relative to the core states.

The inverse participation ratio (IPR)^13^ was calculated to quantify carrier localization (Figure 9):

$$\mathrm{IPR}(E)=\frac{\sum_{i}p_{i}(E{)}^{2}}{{\left[\sum_{i}p_{i}(E)\right]}^{2}}$$ 2

where *p*_*i*_(*E*) is the local density of states for atom *i*, with IPR = 1 indicating full localization and IPR → 0 indicating full delocalization. In the *Cc* ground state, IPR at the VBM is 0.01, indicating essentially complete charge delocalization across N atoms. Disorder increases VBM localization progressively, reaching a maximum of 0.07 at 2000 K. The CBM IPR remains unchanged at synthesis temperatures, consistent with its Ca *d* character and insensitivity to Al/Si disorder. The greatest contribution to states near the VBM comes from Al-rich motifs (N4–30, N5–20), as shown by the partial DOS (Figure S12) and the charge density of the 2000 K supercell.

**Figure 9:**
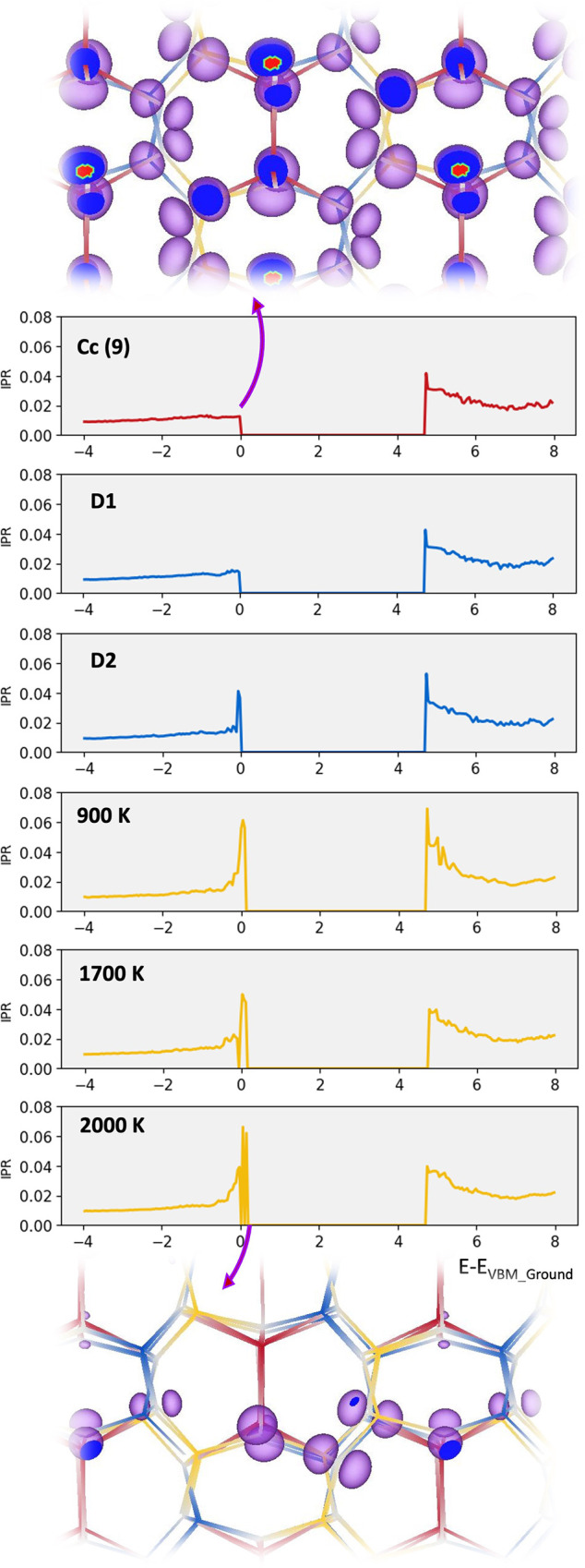
Normalized IPR for the 144-atom supercells. Band edges are aligned relative to the core states. Charge-density diagrams illustrate the degree of localization at VBM of *Cc* and 2000 K structures. Isosurface level is 0.00*8 eV/*Å^3^. Ca, red; Al, yellow; Si, blue; N, gray; and isosurface, purple.

The modest band gap reduction across and lack of charge localization all disordered supercells reflects two features of CASN’s crystal chemistry acting in concert. The CBM is dominated by Ca *d* states and is therefore decoupled from the disordered Al/Si sublattice, bounding the electronic response to disorder. The VBM, composed of N *p* states, shifts upward with increasing disorder, driven by the growing population of Al-rich motifs with bond strength sums below 3+. However, because the disorder is strongly correlated rather than random, these extreme motifs remain a minority even at synthesis temperatures—at 2000 K, over 80% of N4 atoms and 70% of N5 atoms retain their ground-state coordination environments. Together, these two effects account for the electronic robustness of CASN under synthesis-relevant disorder.

## Discussion

Cation disorder in CASN leads to a measurable but modest band gap reduction (−3.8*%* at 2000 K relative to the *Cc* ordering). As discussed above, this resilience has two origins: the CBM is dominated by Ca *d* states and is insensitive to Al/Si configurational disorder, while the strongly correlated nature of the disorder limits the population of electronically unfavorable N motifs. This contrasts with ternary nitrides such as ZnSnN_2_ and ZnGeN_2_, where less correlated disorder directly perturbs both band edges—Zn-rich motifs shift both the VBM and CBM, driving pronounced band gap collapse and carrier localization^11–13^ —because no equivalent disorder-insensitive CBM exists in those systems. The electronic robustness of CASN therefore reflects two features of its crystal chemistry: a CBM decoupled from the disordered sublattice, and a correlated disorder regime that suppresses the most electronically damaging local environments.

The stability of CASN motifs follows from electrostatic descriptors. Bond strength sums of key motifs (N4–21, N4–12, N5–11) lie close to 3+, ensuring approximate charge balance, while even the Pauling-rule-violating motifs (N4–30, N4–03, N5–20, N5–02) show only small deviations (within ±0.4). This is far less extreme than in Zn*X*N_2_, where Zn-rich (4:0) and X-rich (0:4) motifs deviate strongly from 3+, leading to large increases in inverse participation ratio (IPR) at the band edges. Figure S13 shows that the Madelung potential energy distributions broaden with increasing disorder, but the degree of broadening is modest, correlating with the relatively small band edge shifts.

The arrangement of motifs also shapes the disorder–property relationship. Representative disordered structures reveal clustering of Al- and Si-rich motifs, but because the bond strength sums of even the most extreme CASN motifs remain close to 3+, partial segregation has limited electronic consequence. Charge localization driven by electrostatic potential fluctuations is a well-documented consequence of cation disorder in many functional materials,^11,50,57,58^ and in those systems clustering of high-energy motifs is directly detrimental. In CASN, the near-ideal bond strength sums mean that no such high-energy motifs exist in sufficient concentration to drive this behavior.

To isolate the role of short-range order, we analyzed a special quasirandom structure (SQS),^59^ which approximates a statistically random alloy with zero Warren–Cowley parameters (details in the Supporting Information). The SQS cell exhibited band gap reduction of −6.0% relative to the ground-state, driven by an upward shift of the valence band maximum associated with increased carrier localization in Al-rich regions (Figure S15). Analysis of N coordination shows that 76% of N4 atoms and 50% of N5 atoms retain ground-state-like environments. Compared to representative disordered supercells, which show a maximum band gap reduction of −3.8%, the larger reduction in the SQS is consistent with this slight shift in motif concentrations. Importantly, despite the absence of short-range correlations, the SQS reproduces the disorder-tolerant electronic behavior, indicating that such tolerance is governed primarily by the distribution of local nitrogen coordination statistics rather than by pair correlations in the cation sublattice.

## Conclusions

In this work, we used cluster-expansion Monte Carlo simulations combined with hybrid density functional theory to investigate Al/Si disorder in the nitride phosphor host CaAlSiN_3_. We demonstrate that CASN undergoes a first-order order–disorder transition near 850 K, evolving from a *Cc*-ordered ground state into a disordered but nonrandom phase characterized by strong short-range correlations. Because typical synthesis temperatures lie well above this transition, experimentally realized CASN is expected to exist in this correlated disordered regime.

Despite the introduction of substantial cation disorder and the formation of Al- and Si-rich local coordination motifs, the electronic structure of CASN remains remarkably robust. Hybrid-DFT calculations reveal only a modest band gap reduction (up to 3.8%) and limited carrier localization even at disorder levels representative of synthesis conditions. This behavior originates from the preservation of near-ideal bond-strength sums across nitrogen coordination environments and the buffering effect of Ca^2+^, which suppress large electrostatic potential fluctuations that drive electronic degradation in more disorder-sensitive systems. Comparison with a SQS cell, which eliminates short-range correlations, shows similarly disorder-tolerant electronic behavior. This demonstrates that disorder tolerance in CASN is governed primarily by the distribution of local nitrogen coordination motifs, rather than by cation short-range order.

By contrasting CASN with ternary nitrides such as ZnGeN_2_ and ZnSnN_2_, where disorder leads to pronounced band gap collapse and localization, our results establish CASN as a model example of disorder tolerance in a multinary nitride. More broadly, this work highlights that the electronic impact of disorder is not determined solely by its magnitude, but by how local coordination chemistry and electrostatics accommodate configurational complexity. These insights suggest two design principles applicable to functional multinary nitrides and nitride phosphor hosts: a conduction-band minimum decoupled from the disordered cation sublattice, and a local coordination chemistry that enforces near-ideal charge balance and suppresses the most electronically damaging environments even at synthesis-relevant disorder levels.

## Supplementary Material



## Data Availability

Data produced during this work is freely available at 10.25500/1338.

## Notes

Cluster-expansion construction; structural properties; density-of-states and band structures for *P2*_*1*_ ordering; optical absorption for *Cc* structure; density-of-states and Madelung site potential energy distributions for representative supercells; cluster vectors; Pauling’s electrostatic valence rule calculations; and results for special quasirandom structure (PDF)
