# Supplementary material for: Disorder Tolerance in LED Phosphor Host CaAlSiN3: Correlated Cation Disorder with Minimal Electronic Penalty
Source: Chem Mater. 2026 Jul 7;38(14):7179–87. doi: 10.1021/acs.chemmater.6c00669 (PMC13420586; doi:10.1021/acs.chemmater.6c00669)
Supplement: Supplementary file 1 [file cm6c00669_si_001.pdf]

Supporting Information: Disorder Tolerance in LED Phosphor Host $CaAlSiN_3$: Correlated Cation Disorder with Minimal Electronic Penalty

Maryia Shymanovich,[†] Alexander G. Squires,[*,†] Jakoah Brgoch,[‡,¶] and David O. Scanlon[*,†]

†*School of Chemistry, University of Birmingham, Edgbaston, Birmingham B15 2TT, United Kingdom*

‡*Department of Chemistry, University of Houston, Houston, Texas 77204, United States*

¶*Texas Center for Superconductivity, University of Houston, Houston, Texas 77204, United States*

E-mail: a.squires@bham.ac.uk; d.o.scanlon@bham.ac.uk

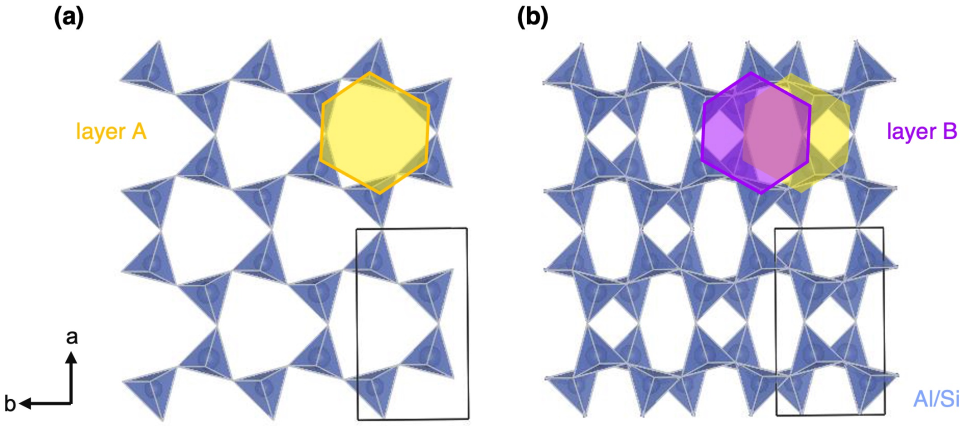

Figure S1: Representation of layers formed by six-membered rings of Al/Si tetrahedra in $CaAlSiN_3$. (a) shows layer A only and (b) shows layer B on the top of layer A. Al/Si - light-blue

Cluster Expansion Construction

The cluster expansion (CE) model was trained using automatic relevance detection regression (ARDR) method. The train set consists of 133 structures. To get an optimum value of cluster cut-off values, the convergence was tested with respect to cut-off radius. **Figure S2** shows the dependence of cross-validated root-mean-square error (CV error), number of non-zero parameters, and predictive coefficient R^2 on cluster cut-off. A pair cut-off of 6.0 Å, which resulted in 16 symmetry-inequivalent pair clusters, low CV error (5.5 meV/atom), and good predictive capability ($R^2 = 0.87$) was chosen for triplet cut-off convergence. At triplet cut-off of 5.0 Å, only two non-zero symmetry-inequivalent triplet clusters were present and gave no significant difference. Above 5.0 Å the CV error increases abruptly.

Predictive capability was tested on another set of 51 structures (validation set). Validation error was calculated using an equation for root-mean-square error[1] (for validation set only) and plotted for a range of pair and triplet cut-off values as shown in **Figure S3**.

Energies predicted by cluster expansion are plotted against DFT-calculated energies in **Figure S4**. The CE model containing pair clusters with 6.0 Å cut-off (**Figure S4(a)**) ac-

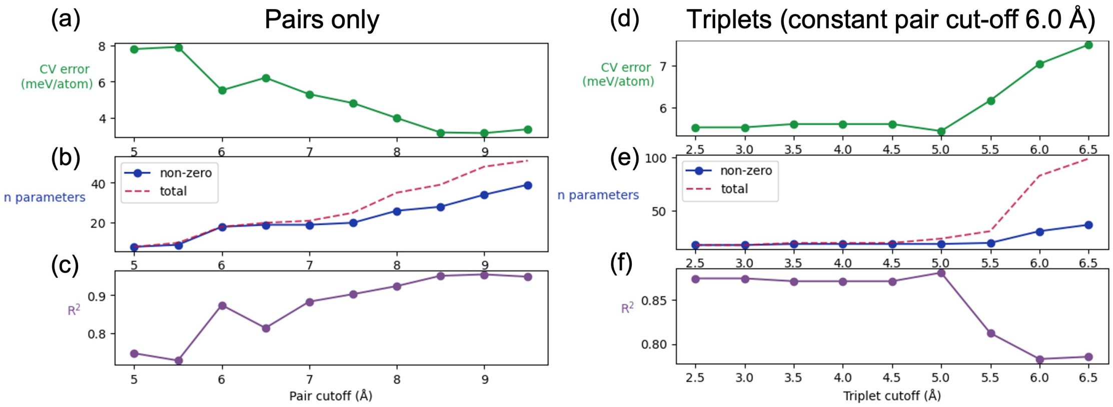

Figure S2: Learning curves showing the dependence of CV error, number of non-zero parameters, and predictive coefficient R^2 on (a-c) pair and (d-f) triplet cut-off radius.

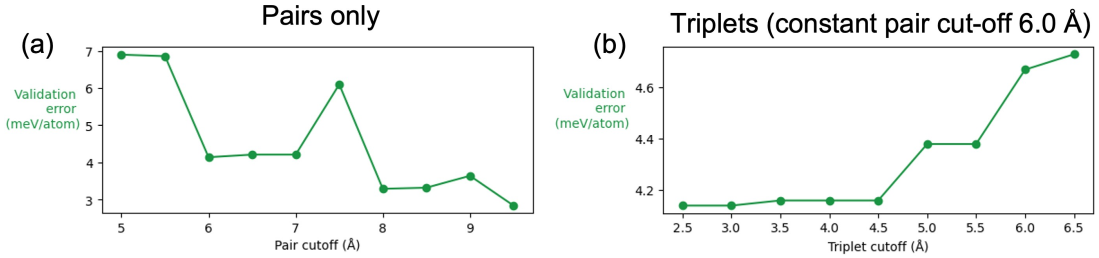

Figure S3: Learning curves showing the dependence of validation error on (a) pair and (b) triplet cut-off radius.

curately predicts the energies of the most CASN structures, including Cc and $P2_1$ orderings. Comparing 6.0 Å with larger pair cutoff of 8.0 Å (**Figure S4(b)**), the latter one results in slightly lower CV and validation errors, however, it incorrectly calculates relative energies of the low-energy structures (below -7.70 eV/atom), predicting wrong ground-state ordering. A triplet cut-off of 5.0 Å (**Figure S4(c)**) has a negligible impact on the predictive accuracy of training and validation sets and, therefore, will not be included in the final CE model.

As shown in **Figure S4(a)**, the predictive error increases significantly for the four high-energy structures from the training set with DFT-calculated energies above -7.66 eV/atom. The relative occurrence probabilities of the CASN configurations can be evaluated by the Boltzmann:[2]

$$P_m = \frac{1}{Z}\Omega_m exp\left(-\frac{E_m}{kT}\right) \tag{S1}$$

Where $m = \{1, \ldots, 133\}$, E_m is the DFT-calculated energy, k is the Boltzmann constant, T is the synthesis temperature of CASN, Ω_m is the multiplicity, and Z is the partition function. At fixed temperature, the probability of occurrence of a configuration decreases exponentially as the energy increases. As a result, the contribution of each configuration to the system decreases with energy, and the larger predictive error of high-energy structures is acceptable.

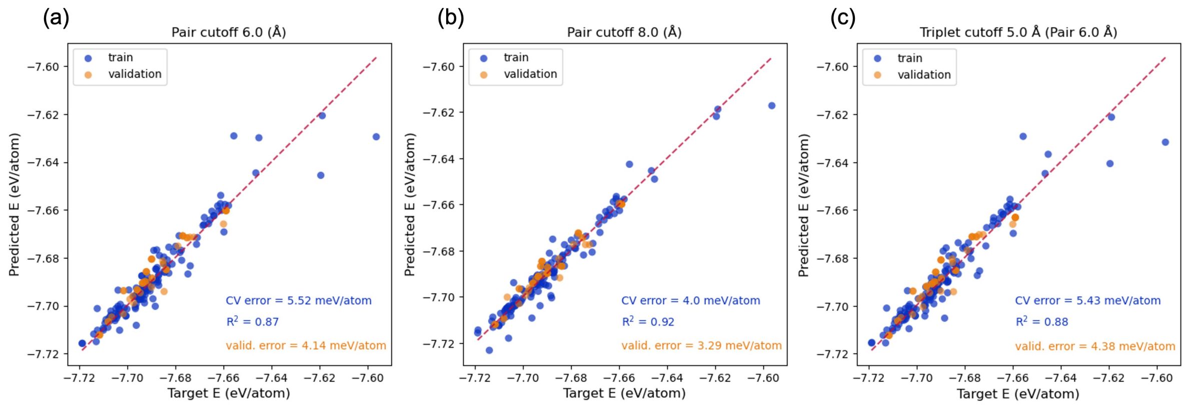

Figure S4: CE-predicted energies of training (blue) and validation (orange) structures compared to those from DFT for the following cut-off basis sets: (a) pair 6.0 Å, (b) 8.0 Å, and (c) pair 6.0 Å + triplet 5.0 Å. CV error and R^2 for the training set is indicated in blue, validation error - in orange.

The CE model that includes 6.0 Å pair clusters only and 133 training set structures served as a compromise between low error and accurate prediction of low-energy configurations and was used as a final model for Monte Carlo simulations.

The 24-atom orderings were also relaxed with hybrid HSE06 functional. The graph in **Figure S5** shows that the total energy values calculated with PBEsol and HSE06 functionals have linear correlation. Both functionals result in the same lowest-energy ordering, which was found to be the ground state among the whole set of structures. So, PBEsol can be used to compute energies for CE model with no need to perform calculations for a large set of structures with more expensive functional.

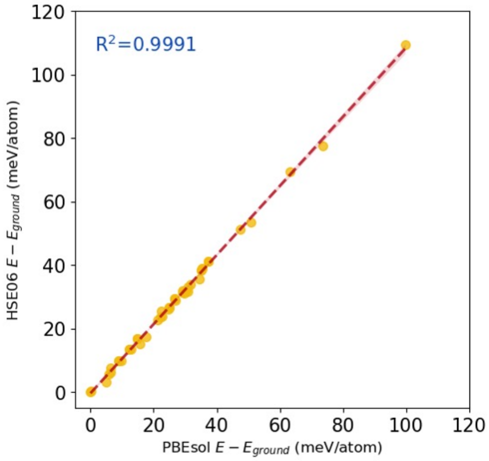

Figure S5: DFT total energies of 24-atom ordered structures of CASN. Energies calculated with PBEsol functional are plotted against energies obtained with HSE06 functional. Red dashed line is the regression line. Coefficient of determination R^2 is given in top-left corner.

Figure S6 gives the effective cluster interactions (ECIs) coefficients for each of the 16 pair clusters in the order of increasing pair distance. The clusters with indices 1-4 correspond to the nearest- neighbouring. The magnitudes of the first four ECIs for these clusters are significantly larger than the rest, which is an indication of strong short-range ordering.

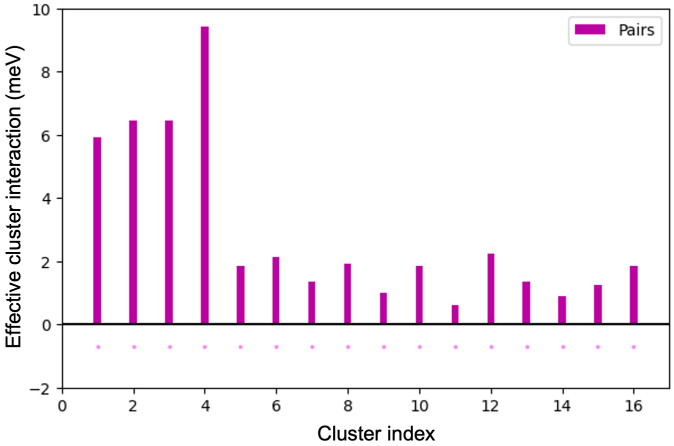

Figure S6: Pair effective cluster interaction (ECI) parameters from CE model. ECIs are displayed in the order of increasing pair distance (1 – shortest pair, 16 – longest pair).

Table S1: Cation-anion bond lengths and average polyhedral bond lengths observed in DFT-HSE06 optimised Cc and $P2_1$ structures, compared with previous theoretical and experimental data.(Distortion indices are obtained from VESTA[5])

<table>
<tr><th></th><th>Cc (9)
this study</th><th>$P2_1$ (4)
this study</th><th>Cc (9)
Mikami et al.[3]</th><th>$P2_1$ (4)
Mikami et al.[3]</th><th>Exp.
Piao et al.[4]</th></tr>
<tr><td colspan="6">Bond length (A)</td></tr>
<tr><td>Si-N4</td><td>1.7219
1.7634
1.7714</td><td>1.7305
1.7566
1.7723</td><td rowspan="4">Si-N
1.737
1.742
1.783
1.787
Al-N
1.880
1.881
1.890
1.907</td><td rowspan="4">Si-N
1.742
1.745
1.773
1.788
Al-N
1.872
1.883
1.889
1.920</td><td rowspan="2">Al/Si-N4
1.7357
1.8009
1.8096</td></tr>
<tr><td>Al-N4</td><td>1.8651
1.8754
1.8934</td><td>1.8575
1.8742
1.9044</td></tr>
<tr><td>Si-N5</td><td>1.7281</td><td>1.7273</td><td rowspan="2">Al/Si-N5
1.8556</td></tr>
<tr><td>Al-N5</td><td>1.8668</td><td>1.8682</td></tr>
<tr><td>Ca-N4</td><td>2.4030
2.4240</td><td>2.4056
2.4203</td><td rowspan="2">Ca-N
2.406
2.432
2.492
2.521
2.603</td><td rowspan="2">Ca-N
2.407
2.424
2.481
2.524
2.599</td><td>2.3995
2.3995</td></tr>
<tr><td>Ca-N5</td><td>2.4958
2.5099
2.5826</td><td>2.4901
2.5147
2.5770</td><td>2.4433
2.5637</td></tr>
<tr><td colspan="6">Average polyhedral bond length (A) {Distortion index}</td></tr>
<tr><td>[AlN_4]</td><td>1.8752
{0.0049}</td><td>1.8761
{0.0075}</td><td>1.900</td><td>1.891</td><td></td></tr>
<tr><td>[SiN_4]</td><td>1.7472
{0.0127}</td><td>1.7466
{0.0101}</td><td>1.762</td><td>1.762</td><td></td></tr>
<tr><td>[CaN_5]</td><td>2.4830
{0.0224}</td><td>2.4815
{0.0221}</td><td>2.491</td><td>2.487</td><td></td></tr>
</table>

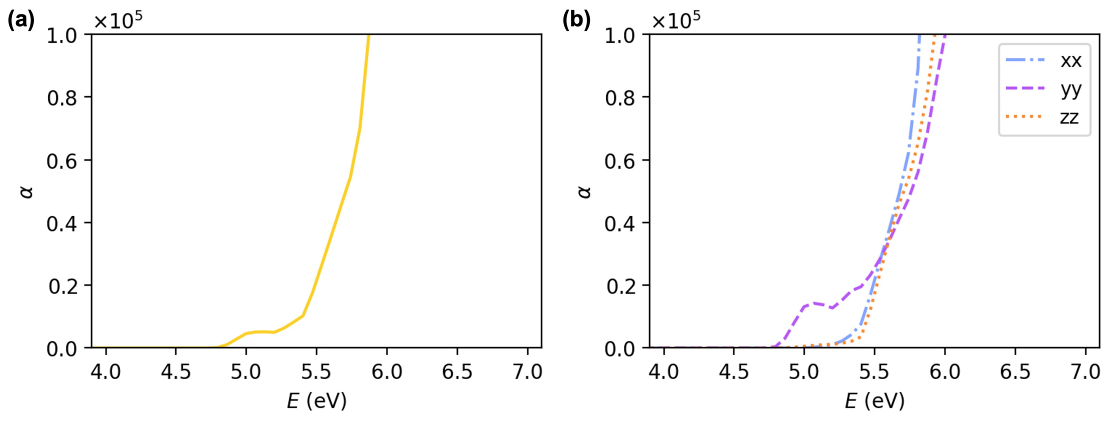

Figure S7: (a) Band-band average optical absorption for ground-state *Cc* structure. (b) Anisotropic contributions from x, y, and z directions.

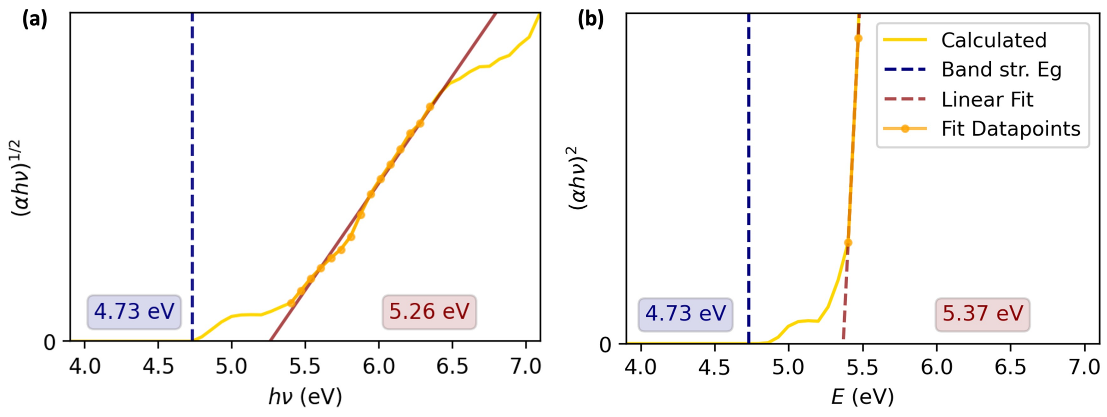

Figure S8: (a) Indirect and (b) direct Tauc fit of the absorption of *Cc* structure. Fundamental (band structure) and optical (intersection point of linear fit) band gap values are given in blue and red boxes, respectively.

Table S2: Coordination (first-nearest-neighbours) and electrostatic bond strength sum, calculated using Pauling's electrostatic valence rule,[6] for each N motif in CASN.

N motif	Coordination	Electrostatic bond strength sum
N4-30	1 Ca^{2+} + 3 Al^{3+}	$\frac{2+}{5} + 3 \times \frac{3+}{4} = 2.65+$
N4-21	1 Ca^{2+} + 2 Al^{3+} + 1 Si^{4+}	$\frac{2+}{5} + 2 \times \frac{3+}{4} + \frac{4+}{4} = 2.90+$
N4-12	1 Ca^{2+} + 1 Al^{3+} + 2 Si^{4+}	$\frac{2+}{5} + \frac{3+}{4} + 2 \times \frac{4+}{4} = 3.15+$
N4-03	1 Ca^{2+} + 3 Si^{4+}	$\frac{2+}{5} + 3 \times \frac{4+}{4} = 3.40+$
N5-20	3 Ca^{2+} + 2 Al^{3+}	$3 \times \frac{2+}{5} + 2 \times \frac{3+}{4} = 2.70+$
N5-11	3 Ca^{2+} + 1 Al^{3+} + 1 Si^{4+}	$3 \times \frac{2+}{5} + \frac{3+}{4} + \frac{4+}{4} = 2.95+$
N5-02	3 Ca^{2+} + 2 Si^{4+}	$3 \times \frac{2+}{5} + 2 \times \frac{4+}{4} = 3.20+$

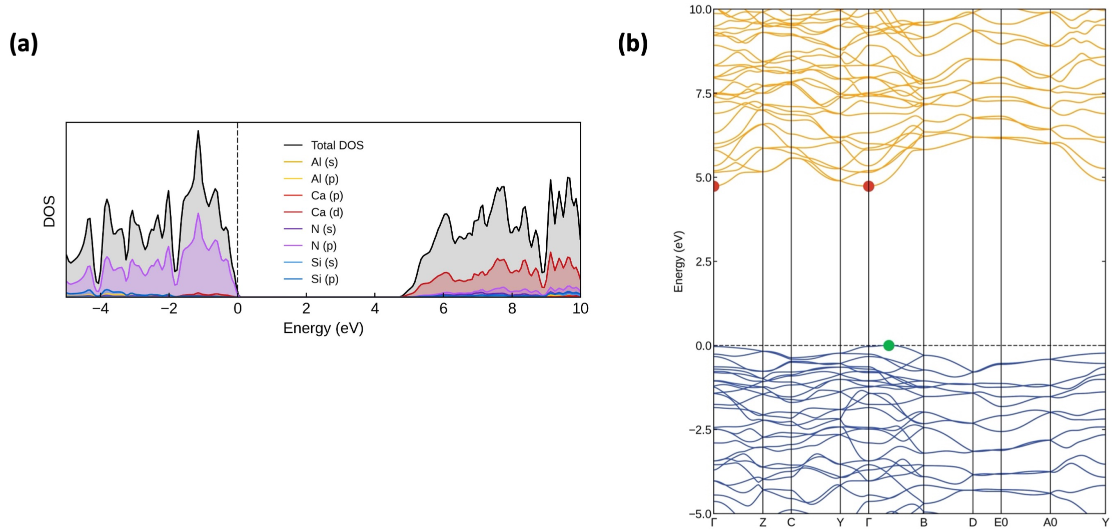

Figure S9: (a) DOS and (b) band structure of $P2_1$ ordered structure. Valence band maximum is used as zero level on both graphs.

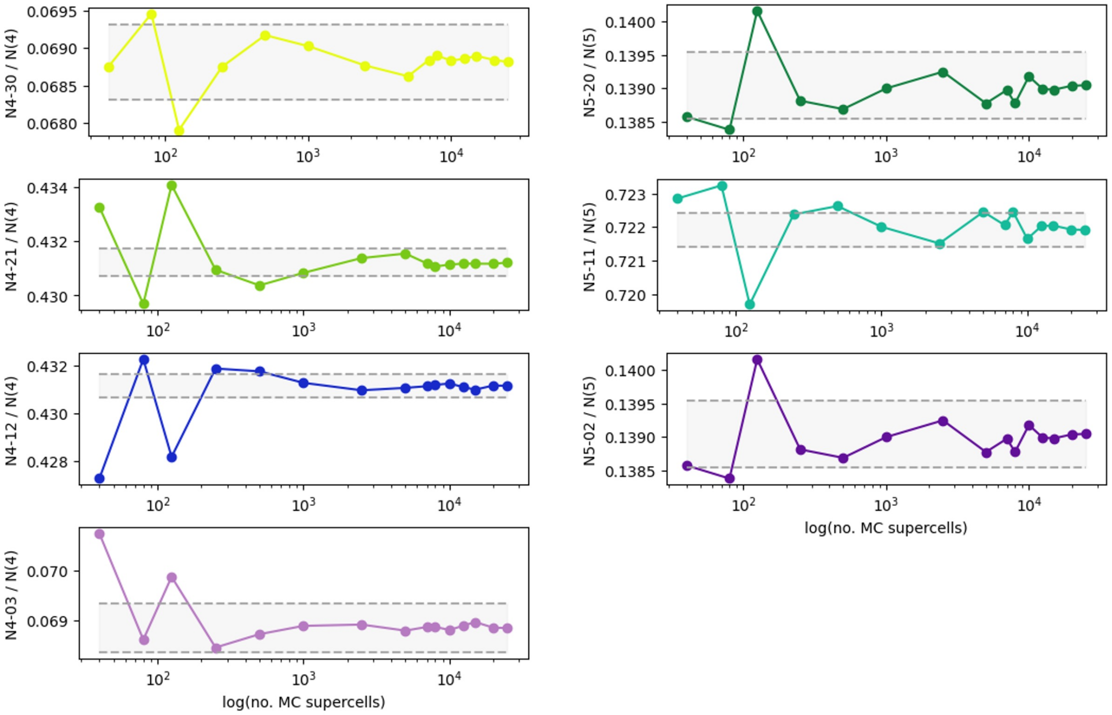

Figure S10: Convergence of the N motif fraction w.r.t. the number of randomly chosen supercells from Monte Carlo simulations. The grey dashed lines display ±0.005 values relative to the largest number of supercells. 1000 supercells were shown to give a well-converged motif fractions for every N local environment.

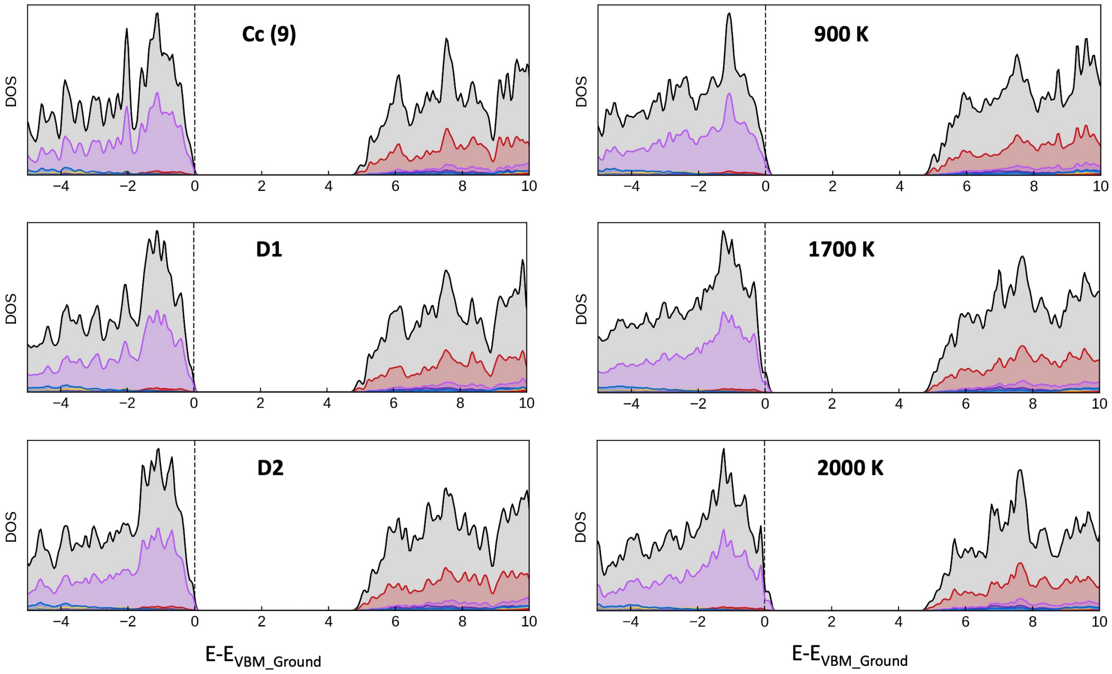

Figure S11: DOS for ordered *Cc* and disordered 144-atom supercells. Zero point of each plot is the VBM of *Cc* structure. VBM are aligned relative to core states.

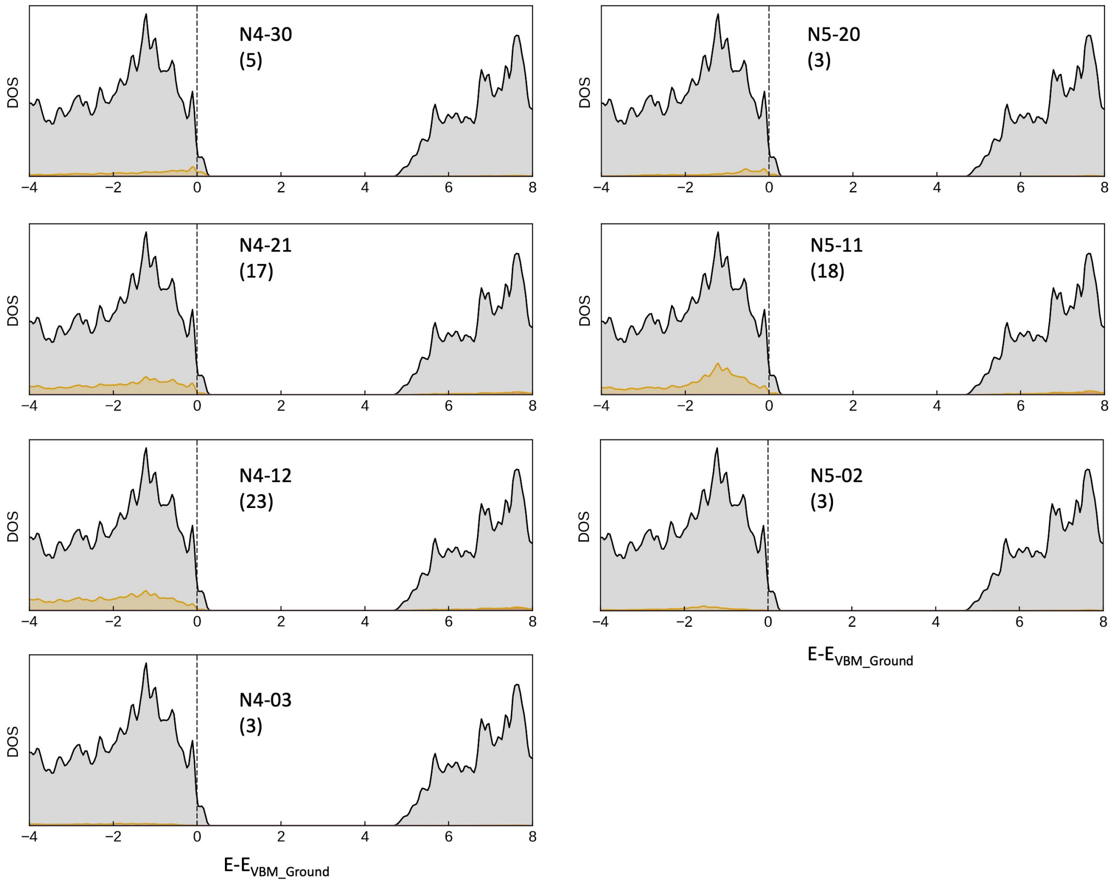

Figure S12: Contribution of N motifs to the DOS of 2000 K disordered supercell. The value in brackets indicate the number of N atoms with the corresponding local environment. Zero point of each plot is the VBM of *Cc* structure.

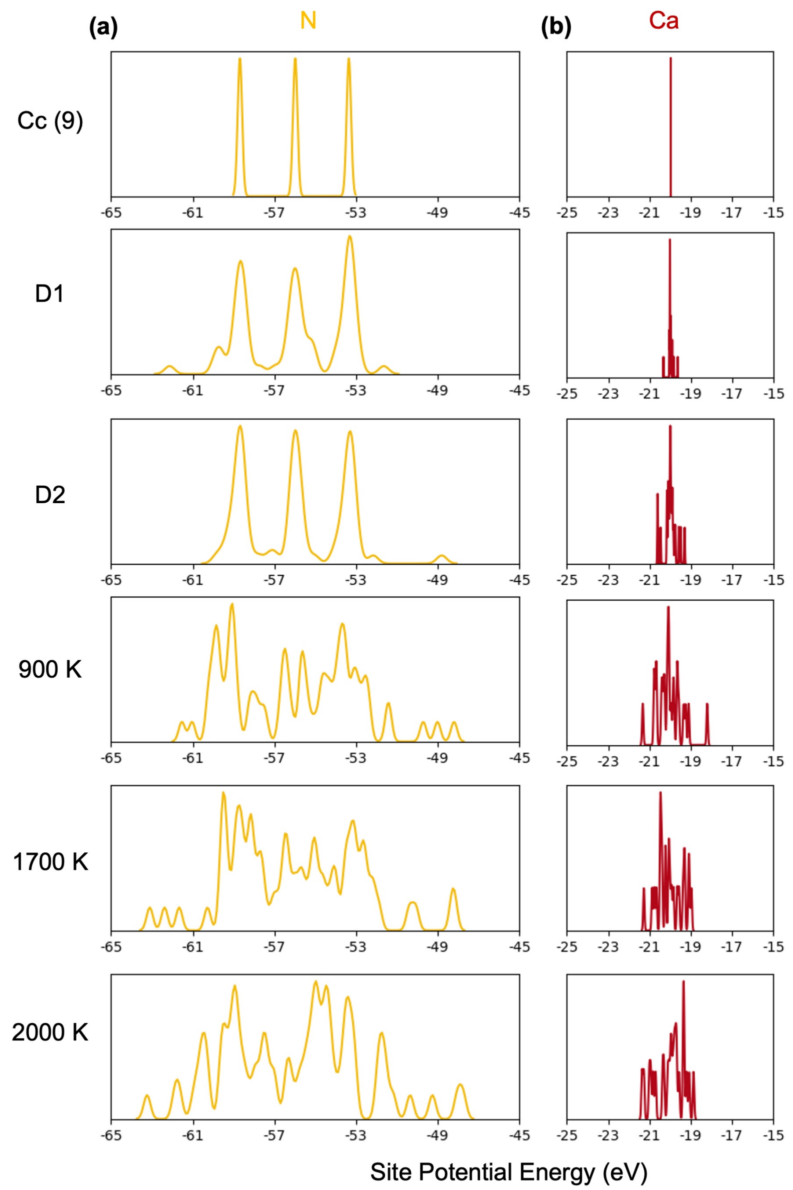

Figure S13: Madelung site potential energy (eV) distributions at (a) N and (b) Ca sites obtained for each of the 144-atom supercells.

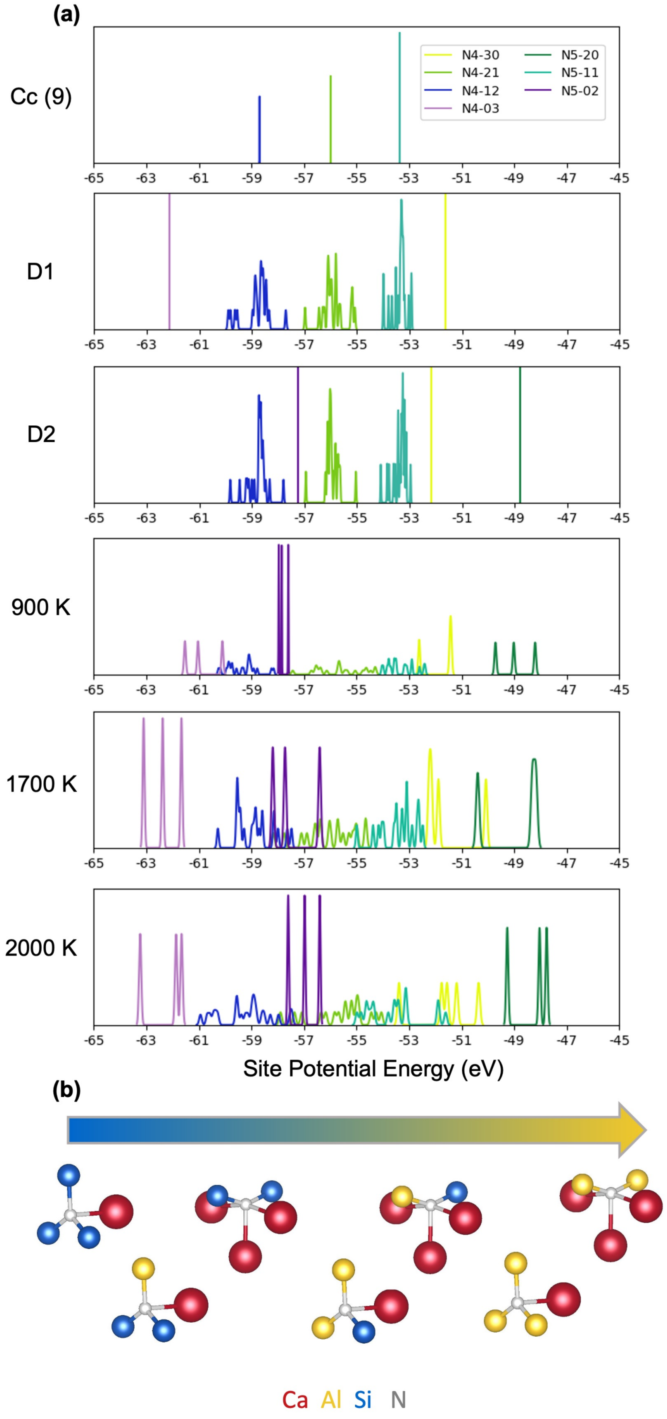

Figure S14: (a) Madelung site potential energy (eV) distributions at N sites plotted separately for each motif type. (b) Schematically shows the correlation between site potential energy and N motif. Al-yellow, Si-blue, Ca-red, N-grey.

Table S3: Comparison of the average pair cluster vectors obtained from Monte-Carlo simulations (MC) with the cluster vectors of 144-atom representative supercells (rep. str.) at 100, 900, 1700, and 2000 K. Cluster vectors of *Cc* and $P2_1$ ordered structures are given. Pair clusters are indexed from 1 to 16, as in **Figure S6**.

	Pair cluster index															
	1	2	3	4	5	6	7	8	9	10	11	12	13	14	15	16
	900 K															
MC	-0.1896	-0.2013	-0.1949	-0.7650	0.1716	0.1062	0.1412	-0.0426	0.1375	0.0976	0.1346	0.0935	-0.0445	-0.0890	-0.0825	-0.1649
rep. str.	-0.1667	-0.1667	-0.1667	-0.5000	0.1667	0.1667	0.1667	-0.0833	0.0833	0.0833	0.0833	0.0833	-0.0833	-0.0833	-0.0833	-0.1667
	1700 K															
MC	-0.1478	-0.1682	-0.1589	-0.5009	0.0892	0.0251	0.0459	-0.0085	0.0538	0.0112	0.0659	-0.0100	-0.0331	-0.0007	-0.0228	-0.0695
rep.str.	-0.0833	-0.1667	-0.1667	-0.5	0.1667	-0.0833	-0.0833	0	0	0.0833	0	0	0	0	0	0
	2000 K															
MC	-0.1352	-0.1612	-0.1531	-0.4437	0.0675	0.0177	0.0356	-0.0089	0.0427	0.0031	0.0565	-0.0183	-0.0259	0.0030	-0.0195	-0.0600
rep. str.	-0.0833	-0.0833	-0.1667	-0.5	0	0	0.0833	0	0	0	0.0833	0	-0.0833	0	0	-0.0833
	Cc															
	1	-1	-1	-1	-1	1	1	-1	1	1	1	1	1	-1	-1	-1
	$P2_1$															
	-1	1	-1	-1	1	-1	1	1	-1	-1	1	1	1	1	-1	-1

Special quasirandom structure

Special quasirandom structure (SQS)[7] was generated using *icet*.[1] The resulting SQS is a 96-atom structure (**Figure S15 (a)**). Its energy is +39 meV/atom relative to the Cc, as calculated with HSE06 functional. Its pair cluster vectors resemble the cluster vectors of statistically random alloy. Warren-Cowley SRO parameter for each pair cluster is equal to zero. SQS displays band gap of 4.46 eV, obtained from band structure calculations with HSE06. The concentrations of N4-21 and N4-12 are 0.281 and 0.479, respectively. The concentrations of N4-30 and N4-03 motifs are 0.156 and 0.094, respectively. The concentration of N5-11 is 0.5; N5-20 and N5-02 are equal to 0.25.

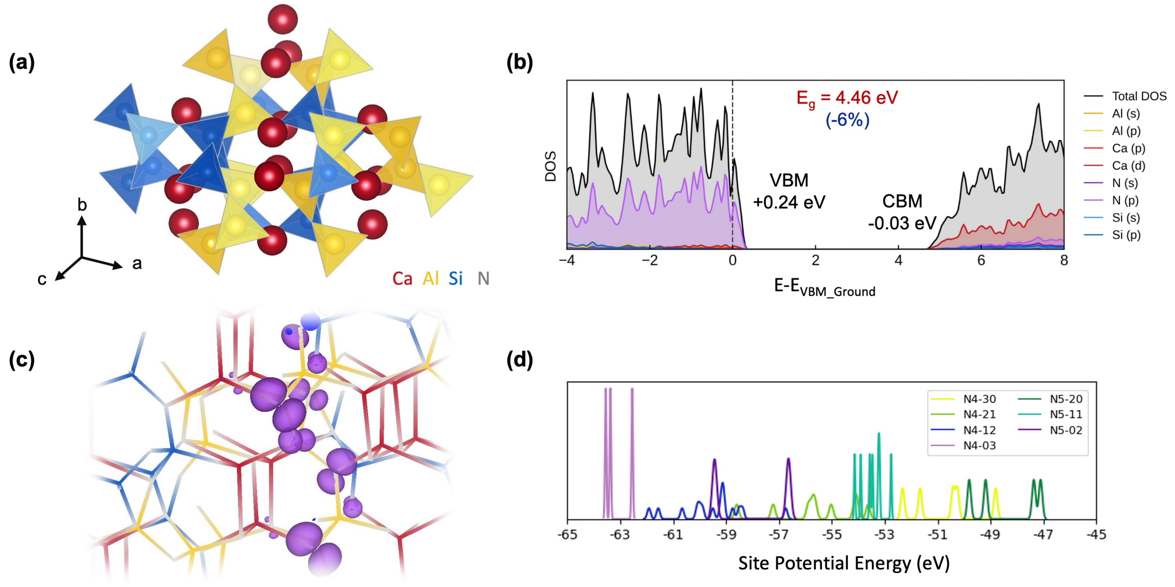

Figure S15: Results for SQS supercell of $CaAlSiN_3$. (a) 96-atom cell of SQS. Ca atoms and Al/Si tetrahedra are shown. (b) DOS of SQS; VBM is aligned relative to core states, zero is the VBM of Cc. Size of band gap is shown in red, reduction relative to Cc in blue. Band edges shifts w.r.t. Cc are given in black. (c) Charge density at of the highest-occupied state (VBM) showing localization at Al-rich region. Isosurface level is set to 0.008 $eV/Å^3$. (d) Madelung site potential energy (eV) distributions at N sites plotted separately for each of the seven N motifs. Al-yellow, Si-blue, Ca-red, N-grey.

References

(1) Ångqvist, M.; Muñoz, W. A.; Rahm, J. M.; Fransson, E.; Durniak, C.; Rozyczko, P.; Rod, T. H.; Erhart, P. ICET – A Python Library for Constructing and Sampling Alloy Cluster Expansions. *Advanced Theory and Simulations* **2019**, *2*, 1900015.

(2) Landau, L. D.; Lifshitz, E. M. *Statistical Physics. Part I*, 3rd ed.; Pergamon Press: Oxford, 1980; Vol. 5.

(3) Mikami, M.; Uheda, K.; Kijima, N. First-principles study of nitridoaluminosilicate CaAlSiN3. *Physica Status Solidi (A) Applications and Materials Science* **2006**, *203*, 2705–2711.

(4) Piao, X.; Machida, K. I.; Horikawa, T.; Hanzawa, H.; Shimomura, Y.; Kijima, N. Preparation of CaAlSiN3:Eu2+ phosphors by the self-propagating high-temperature synthesis and their luminescent properties. *Chemistry of Materials* **2007**, *19*, 4592–4599.

(5) Momma, K.; Izumi, F. VESTA: A three-dimensional visualization system for electronic and structural analysis. *Journal of Applied Crystallography* **2008**, *41*, 653–658.

(6) Pauling, L. *The Nature of the Chemical Bond and the Structure of Molecules and Crystals*, 3rd ed.; Cornell University Press: New York, 1939; Chapter 13.6, pp 543–562.

(7) Zunger, A.; Wei, S.-H.; Ferreira, L. G.; Bernard, J. E. Special Quasirandom Structures. *PHYSICAL REVIEW LETTERS* **1990**, *65*, 353–356.
